# Thyroid Storm: A Case Report

**DOI:** 10.7759/cureus.84694

**Published:** 2025-05-23

**Authors:** Carolina António Santos, Ana Filipa Vassalo, José Rocha, Ana Alves Cardoso, Frederico Trigueiros

**Affiliations:** 1 Internal Medicine, Unidade Local de Saúde Santa Maria, Lisbon, PRT; 2 Immunoallergology, Unidade Local de Saúde Santa Maria, Lisbon, PRT; 3 Endocrinology, Unidade Local de Saúde Santa Maria, Lisbon, PRT; 4 Medicine, Associação Protectora dos Diabéticos Portugueses, Lisbon, PRT

**Keywords:** anemia, graves´ disease, heart failure, liver failure, thyroid storm

## Abstract

Thyroid pathology is quite common worldwide. Thyroid storm is a severe form of hyperthyroidism, particularly in Graves' disease, which is rare. It is in association with various triggering factors (therapy discontinuation, infections, surgical interventions) and can lead to multiorgan dysfunction, particularly liver and cardiovascular injury. Prompt diagnosis and early therapy are fundamental to solving the thyroid storm and its complications. Multiple therapies are needed for adjustment according to the particular case, including the use of synthetic antithyroid drugs, beta-blockers, corticosteroids, and supportive therapies, depending on the systems affected. This article reports a case of long-standing Graves' disease that culminated in an episode of thyroid storm with multiorgan dysfunction (45 points on the Burch-Wartofsky point scale), representing a diagnostic challenge. Multiple therapies were needed to reach the euthyroid state, after which there was a complete resolution of the entire condition.

## Introduction

Thyroid pathology is common globally [1,2] and more frequently seen in women [1,3], with a prevalence of hyperthyroidism estimated at between 0.2-1.3% [1]. Thyroid storm (TS) is a rare and potentially fatal form of thyrotoxicosis [2,4-6] (16% of inpatients with thyrotoxicosis were diagnosed with TS according to US data) [5], which can be associated with multiple triggers [4-6]: discontinuation of synthetic antithyroid therapy (ATD) [4-6], acute events [5] (surgery [4-6], trauma [4-6], infection [4,5], acute cardiovascular pathology [6], burns [6], childbirth [4-6]), high iodine levels [4,5], or iatrogenesis (e.g. amiodarone) [4,6]. It can have different etiologies, with Graves' disease (GD) being the most common [4-6], accounting for 50-80% of cases of hyperthyroidism [1]. Prompt diagnosis and early initiation of therapy are necessary and should not be delayed by etiological investigation [5]. According to the Japan Thyroid Association, clinical and laboratory findings suggest the presence of hyperthyroidism [7]. For the diagnosis of GD, a patient may fulfill at least one clinical criterion (signs of thyrotoxicosis, diffuse enlargement of the thyroid gland, or ophthalmopathy) and the four laboratory criteria (elevation of free thyroxine (fT4) and/or free triiodothyronine (fT3) levels; thyroid-stimulating hormone (TSH) < 0.1uU/mL; positive thyrotropin receptor antibodies (TRAb) or thyroid-stimulating antibody (TSAb) [7]; elevated radioactive iodine uptake to the thyroid gland) [7]. In addition to the exacerbation of thyrotoxicosis symptoms, excess thyroid hormone (TH) in untreated hyperthyroidism [3,6] can lead to multi-organ dysfunction [3,6], affecting the cardiovascular system [2-6] and causing liver damage [2-4,8]. In line with this multiorgan involvement, the Burch-Wartofsky point scale (BWPS) helps predict the likelihood of TS in patients, and laboratory results are compatible with thyrotoxicosis [9]. It evaluates seven signs/symptoms and their severity, strongly suggesting the presence of TS when BWPS > 45 points. This article aims to highlight some less common manifestations of hyperthyroidism based on a clinical case.

## Case presentation

This is a 55-year-old man from Guinea-Bissau, where he resides. He is independent in daily activities and has a history of heart failure (HF), with multiple hospital admissions reported in his home country in this context. His current daily medication includes spironolactone 25 mg, furosemide 40 mg, captopril 25 mg, and digoxin 0.25 mg.

Upon arriving in Portugal, he presented to the emergency department (ER) with a progressively worsening condition, including fatigue on minimal exertion, weight loss, diarrhea (unspecified characteristics), heat intolerance, hypersudoresis, and palpitations. He also reported dyspnea on exertion, tightness-type retrosternal pain lasting over 15 minutes and resolving spontaneously, triggered by exertion but occasionally occurring at rest, along with lower limb (LL) edema and increased abdominal distension. He denied orthopnea, paroxysmal nocturnal dyspnea (PND), syncope, fever, anorexia, tremors, and abdominal pain. 

**Figure 1:**
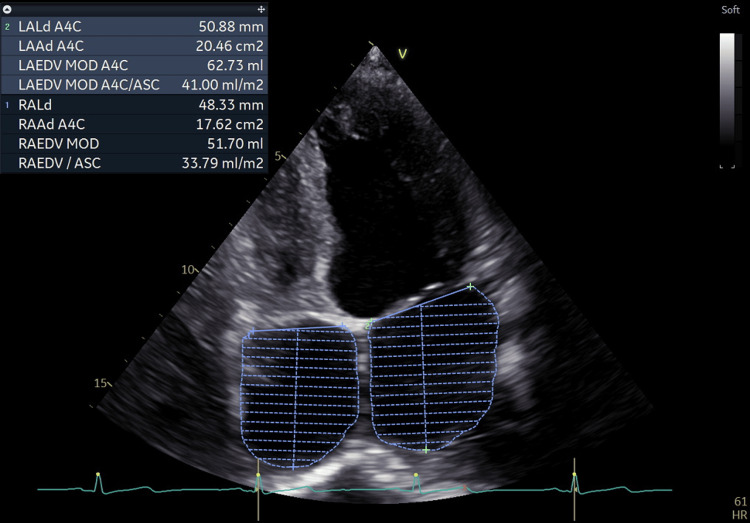
Transthoracic echocardiogram showing the biatrial dilation

On admission, he was afebrile, normotensive (120/61 mmHg), and tachycardic (115 bpm), with a normal respiratory rate on room air. He appeared sarcopenic and dehydrated, with icteric sclerae. Physical examination revealed jugular venous engorgement (JVE), abdominal distension with a positive fluid wave sign, and edema of the LL godet++ up to the knees. Laboratory tests (Table 1, T0) showed normocytic normochromic anemia, no parasitized erythrocytes, and a negative Plasmodium antigen test. Findings included conjugated hyperbilirubinemia, elevated NT-proBNP, increased free T3 (fT3) and free T4 (fT4), a markedly decreased TSH level, and positive anti-thyroperoxidase (anti-TPO), anti-thyroglobulin (anti-TG), and anti-receptor TSH (TRAb) antibodies. A transthoracic echocardiogram (TTE) revealed diffuse left ventricular hypokinesia (Video 1), marked biatrial dilatation (Figure 1), and severe aortic (Video 2), mitral (Video 3), and tricuspid regurgitation (Video 4), with an estimated pulmonary arterial systolic pressure (PASP) of 36 mmHg. BWPS was calculated, with a total score of 45 points, suggesting an established TS.

**Table 1:** Laboratory evolution from admission to one year after radioactive iodine therapy (T represents the time in months since admission to the ER) Hb: hemoglobin; AST: aspartate aminotransferase; ALT: alanine aminotransferase; FIB-4: fibrosis-4 index; GGT: gamma-glutamyl transferase; aPTT: activated partial thromboplastin time; INR: international normalized ratio; NT-proBNP: N-terminal pro–B-type natriuretic peptide; TSH: thyroid-stimulating hormone; fT3: free triiodothyronine; fT4: free thyroxine; Anti-TG: anti-thyroglobulin antibodies; Anti-TPO: anti-thyroid peroxidase antibodies; TRAb: thyrotropin receptor antibodies

Test	Admission (T0)	Discharge (T2)	Readmission (T5)	1 month after radioactive iodine (T7)	1 year after radioactive iodine (T18)	Range
Hb (g/dL)	4.1	8.9	9.0	12.6	12.3	13.0-17.5
Mean corpuscular volume (fL)	86.9	98.6	92.5	91.2	91.6	80.0-97.0
Mean corpuscular hemoglobin (pg)	31.1	32.6	30.5	30.1	30.5	27-33
Leucocytes (x10^9^/L)	10.7	8.50	5.60	7.90	7.60	4.0-11.0
Platelets (x10^9^/L)	159	167	136	304	179	150-450
AST (U/L)	20	34	23	21	22	0-40
ALT (U/L)	13	28	10	15	14	0-41
FIB-4	1.92	2.12	2.94	0.98	1.81	
GGT (U/L)	133	424	650	496	175	0-60
Alkaline phosphatase(U/L)	80	237	288	199	162	35-105
Total bilirrubin (mg/dL)	14.62	1.45	0.73	0.35	0.35	<1.2
Direct bilirrubin (mg/dL)	11.22	1.04	0.43	-	0.12	<0.2
Prothrombin time (seg)	15.6	-	13.4	12.2	11.8	11.6
aPTT (seg)	26.9	-	31.8	35.2	30.2	29
INR	1.35	-	117	1.07	1.04	
Fibrinogen (mg/dL)	135	-	229	400	305	200-400
Ferritin (ng/mL)	2864	-	1082	-	848	30-400
NT-proBNP(pg/mL)	10236	6010	5194	2039	202	<900
TSH (uU/mL)	0.010	<0.005	<0.005	0.010	0.146	0.30-4.20
fT3 (pg/mL)	10.30	6.99	7.67	-	4.09	2.0-4.4
fT4 (ng/dL)	7.27	2.00	1.97	0.85	1.37	0.85-1.70
Anti-TG (U/mL)	21	20	17	-	22	<115
Anti-TPO(U/mL)	103	83	94	-	17	<37
TRAb (U/L)	160	173.50	196.50	-	59.00	<1.23

**Video 1:** Transthoracic echocardiogram revealed diffuse left ventricular hypokinesia

**Video 2:** Transthoracic echocardiogram revealed aortic regurgitation

**Video 3:** Transthoracic echocardiogram revealed severe mitral regurgitation

**Video 4:** Transthoracic echocardiogram revealed tricuspid regurgitation

**Figure 2:**
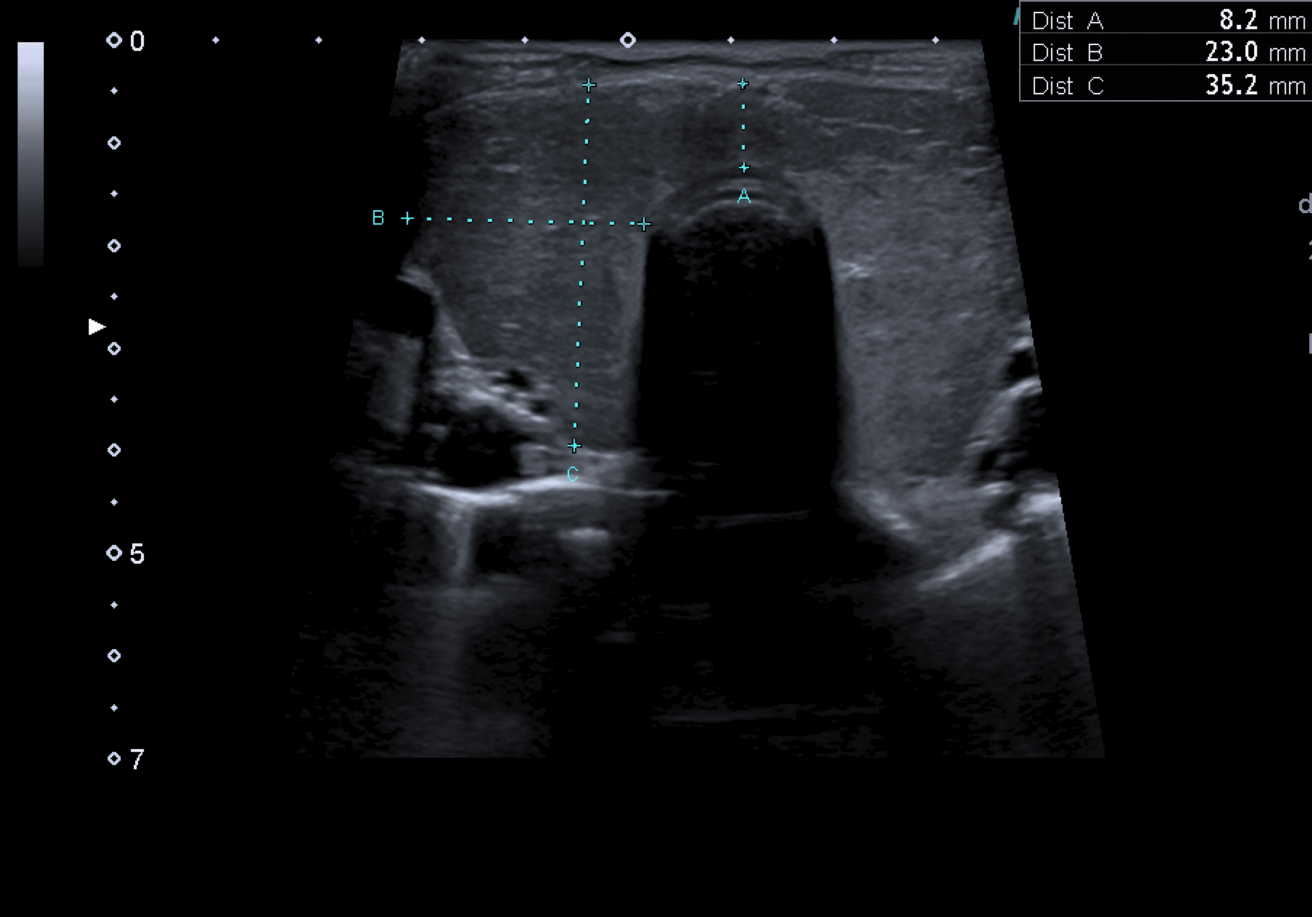
Thyroid ecography showing an enlargement of the gland, without nodules

**Figure 3:**
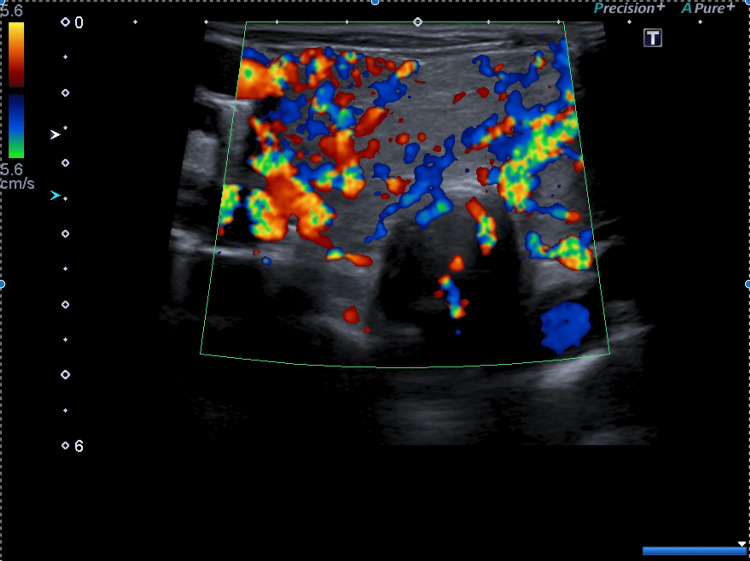
Ecography with Doppler study showing bilateral hypervascularization

He received a transfusion of three units of erythrocyte concentrate and underwent paracentesis (ascitic fluid with alpha-fetoprotein <0.9 ng/mL; 61.3% polymorphonucleated cells; negative microbiological tests). Diuretics were initiated, and, based on a presumed diagnosis of TS due to GD, treatment was started with Lugol solution 5 drops 8/8h, thiamazole 20mg 12/12h, and bisoprolol 5mg 24/24h. He was subsequently admitted to an internal medicine ward. A thyroid ultrasound showed an enlarged gland, heterogeneous parenchyma, no nodules, and bilateral hypervascularization on Doppler study, confirming the diagnosis (Figures 2, 3).

ATD was continued, later transitioning to a block-and-replace (BR) strategy, thiamazole 20 mg/day, and levothyroxine 0.05 mg/day. 

**Figure 4:**
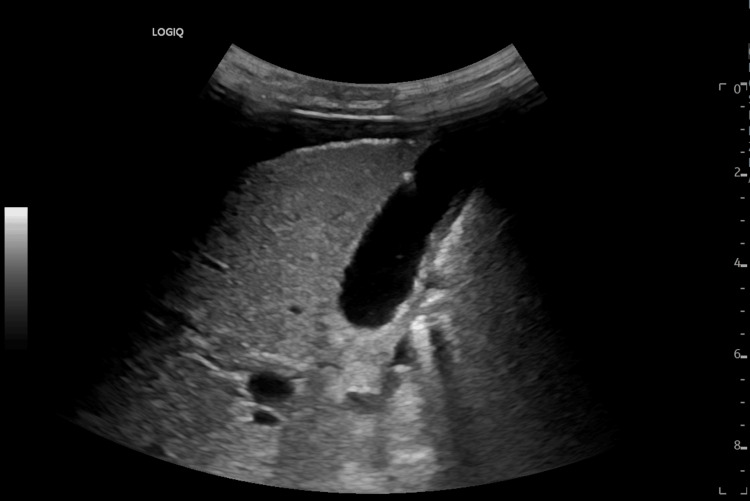
Abdominal ecography without gallbladder alterations and without hepatic focal lesions or alterations in hepatic parenchyma

Although the diagnosis of TS could explain the HF, liver failure, and anemia, other etiologies were ruled out. Given the signs of liver disease and the results of the paracentesis, a serum-ascitic albumin gradient >1.1 supported an elevated hydrostatic pressure in the portal system, suggestive of chronic liver disease (CLD). Imaging studies ruled out alterations to the gallbladder and bile ducts, focal liver lesions (Figure 4), or vascular alterations, excluding other causes of liver dysfunction. Other etiologies of CLD were ruled out: negative viral serologies (hepatitis A, B, C, D, and E; human immunodeficiency virus; cytomegalovirus; Epstein-Barr virus; parvovirus; adenovirus; and herpes simplex virus 1 and 2), negative mycobacteria and intestinal parasite tests, and autoimmune study without alterations except for anti-thyroid antibodies and TRAbs, as mentioned above. Other pathologies were excluded due to non-compliance with diagnostic criteria: alpha-1-antitrypsin deficiency, hemochromatosis, Wilson's disease, and serum amyloidosis. A transjugular liver biopsy revealed a cholestatic lesion, with no signs of portal hypertension. These results, along with the favorable clinical and laboratory evolution under ATDs alone, reinforced the diagnosis of CLD secondary to untreated long-term hyperthyroidism.

From a cardiac perspective, he showed significant improvement with diuretic and hyperthyroidism therapy, leading to the resolution of the congestive condition. The reassessment TTE carried out on the 10th day of hospitalization revealed non-dilated heart cavities, preserved ejection fraction, and hypokinesia of the septum and inferior wall, reinforcing the hypothesis of dilated cardiomyopathy and consequent HF secondary to prolonged untreated hyperthyroidism. 

In terms of hematology, anemia due to iron deficiency, hemolytic anemia, blood loss, and alterations in erythropoiesis were ruled out. Myelogram showed normocellular bone marrow. Initially, he required recurrent transfusional support, but his condition stabilized once hyperthyroidism was controlled.

He was discharged asymptomatic with improved thyroid function (Table 1, T2) and continued follow-up at the internal medicine clinic. However, one month after discharge, he developed progressively worsening fatigue, LL edema, increased abdominal distension, and recurrent tachycardia. Laboratory tests once again showed hyperthyroidism and a hepatic cholestasis pattern (Table 1, T5). Although access to therapy was provided, suspicion of treatment non-compliance was raised due to poor understanding of the therapeutic regimen, justifying his readmission to the hospital for further evaluation and therapeutic optimization.

**Figure 5:**
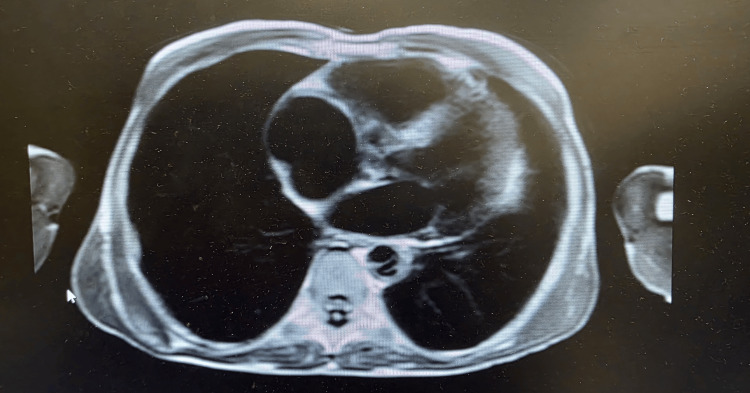
Cardiac magnetic resonance showing dilatation of the cavities, particularly the tricuspid valve ring

**Figure 6:**
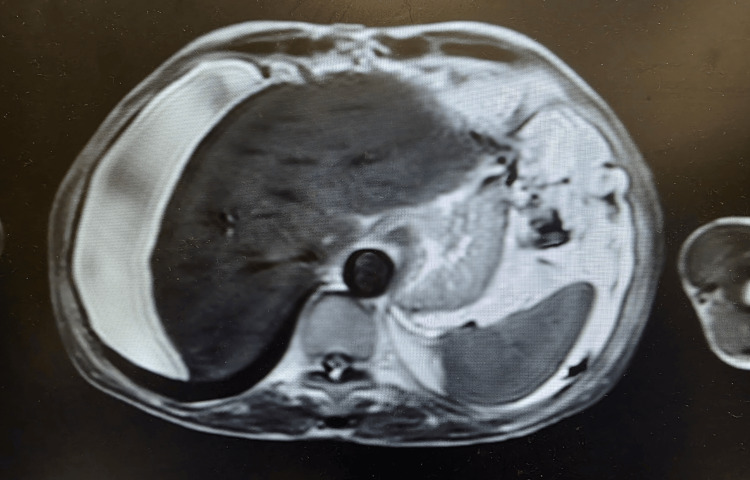
Abdominal magnetic resonance showing a liver without focal lesions and a moderate quantity of ascites

The complementary diagnostic tests carried out included thyroid scintigraphy, which showed intense and diffuse uptake, consistent with a diffusely hyperfunctioning gland; cardiac magnetic resonance imaging revealing slight dilation of the cardiac cavities (Figure 5), with globally preserved ventricular systolic function (Video 4), and slight tricuspid insufficiency due to dilation of the respective valvular ring; and liver imaging suggestive of CLD and significant ascites (Figure 6), corroborating the clinical findings on admission. His cardiac and hepatic affection was once again attributed to uncontrolled hyperthyroidism. Therefore, given the severity of the condition and in order to definitively control the disease, he underwent radioactive iodine therapy (10 mCi), subsequently maintaining the BR therapeutic regimen (thiamazole 15 mg/day and levothyroxine 0.05 mg/day), with clinical and laboratory improvement following treatment (Table 1, T7-T18).

## Discussion

The diagnosis of TS is based on very low/undetectable TSH levels and high concentrations of fT4 [4-6,10] and/or fT3 [4-6] (with no direct correlation between serum TH levels and the clinical severity) [5,6], as well as signs and symptoms of severe hyperthyroidism [2,3,5,6], namely hyperpyrexia [5,6], tachycardia [2,3,5,6], congestive HF [2,3,5,6,11], signs of central nervous system involvement [3-6] (agitation, anxiety [5,6,11], delirium [5,6]) and gastrointestinal manifestations [3-6,11]. The main pathophysiological hypotheses point to a rapid increase in plasmatic levels of TH, hypersensitivity to catecholamines, or an exacerbated cellular response to TH [5,6]. As portrayed in this clinical case, excess TH in untreated hyperthyroidism [3,6], particularly in GD [12], can lead to multi-organ dysfunction [3,6,12], particularly hepatic, cardiovascular, and hematological.

Alterations in liver tests are common [2,3,8,12], generally mild, and asymptomatic [8,12]. In this case, there was an increase in alkaline phosphatase and gamma-GT in both hospital admissions, as well as hyperbilirubinemia in the first admission, with no associated symptoms. The underlying pathophysiological mechanism remains uncertain [2,3,8], and there are several hypotheses [3,4,8]: indirect mechanisms [3,4] (hemodynamic dysfunction due to congestive HF [3,4,8,13-15], hepatocyte anoxia [3,4,8,13,15] due to the production of free radicals [4,13,15] in a state of hypermetabolism [3,4,13], hepatic cell degeneration due to increased protein and glycogen degradation [4,13], the action of TRAbs on the liver [3,4,8,13,15]), the direct action of HT on the liver [3,4,8,13]), autoimmune mechanisms [3,4,8,13,14], or iatrogenesis [4,8,13-15] associated with the use of iodized solutions [6]. Elevated alkaline phosphatase in hyperthyroidism is particularly frequent [2,3,8,11,12] due to the increase in osteoblastic activity [2,3,11] leading to a predominance of the bone isoenzyme [2,3]. Another common change includes a cytocholestatic pattern [2,3,12] with an increase in transaminases [2,3,8,11-14] and gamma-GT [2,3,8,14]. The patient's age, the prolonged duration of the condition (> 1 year) [12], being male, and being of African descent [13] are also factors that may have contributed to hepatic alterations and hyperbilirubinemia [12,14], which generally regress after treatment of hyperthyroidism [2,3,8,12,13] and do not contraindicate the use of ATDs [2,3,8,13].

Cardiac manifestations are also frequent in GD, caused by the positive chrono and inotropic effects [10,11,15,16] of TH on the heart muscle and blood vessels [10,13,16], increasing heart rate [2,3,10,11,15-17] and contractility [10,11,15,16] and beta-agonist effects, resulting in increased cardiac output [10,11,15,16,18], systolic hypertension [6,11,15,16] with wide pulse pressure [10,11,15-17,18], cardiac remodeling with myocyte hypertrophy and dilated cardiomyopathy [10,15], often leading to valvular insufficiency [11,16] (mitral [11,16] or tricuspid [16]), dysrhythmias [6,10,16] and decreased peripheral vascular resistance [10,11,15,16,18]. Thus, there is a greater risk of atrial fibrillation [2,3,10,11,15,16,18], pulmonary hypertension [10,11,15,16], angina [16], and congestive HF [2,3,6,10,11,15,16,18], classic signs and symptoms evident in patients with hyperthyroidism and also present in this case. The patient had a history of HF, with fatigue on slight exertion, dyspnea, and chest pain on exertion, and biatrial dilatation and severe aortic, mitral, and tricuspid regurgitation were documented on TTE. 

From a hematological perspective, GD most often manifests with normocytic normochromic anemia, as described in this case, which is attributed to an increase in erythrocyte mass and blood volume [11,19] and a decrease in erythrocyte half-life [19], constituting a form of anemia of chronic disease [19]. Additionally, elevated ferritin levels were observed [11], reflecting its role as an acute-phase marker. Autoimmune mechanisms and direct effects of TH on hematopoietic cells can also lead to hemolytic anemia [11,19] and pancytopenia with normal/hypercellular bone marrow on myelogram [19]. Thionamides should be used with caution in these patients due to the risk of agranulocytosis [19]. However, in GD, as seen in this case, hematological changes resolve once thyroid dysfunction is controlled, so their use seems to be beneficial [19].

The aims of TS therapy are clinical stabilization [4-6,15,17,18], symptomatic control [4,5,10,15-18], correction of precipitating factors [5,6], and reversal of systemic complications [4,5]. In the initial phase, treatment typically involves four main pharmacological groups: 

Thionamides, which inhibit TH [4-6,17] synthesis. Propylthiouracil is an initial choice in more severe cases, as it concomitantly inhibits the peripheral conversion of T4 into T3 [4-6], while thiamazole is often chosen for its longer-lasting effect [5,6,17], lower hepatotoxicity [5,6], and suitability for outpatient use [5]. In this case, thiamazole was chosen due to the risk of therapy noncompliance and the liver changes observed.

Iodine solutions (in this case Lugol's solution) [4,5,8] can be used after the administration of thionamides [4-6,17] to block the release of TH [4-6,8,17].

Glucocorticoids [4-6,17] (preferably hydrocortisone) [5], which reduce the conversion of T4 into T3 [4-6,8,17], have a potential immunosuppressive effect in immune-mediated diseases [5] such as GD [5,12] and may help manage possible adrenal insufficiency induced by the condition [4,5]. However, there are still no randomized studies confirming the superiority of glucocorticoids over placebo, and their role in survival improvement is still controversial. Therefore, this therapy may be considered case-by-case basis. In this case, due to a hypertensive state and hyperglycemic profile, glucocorticoids were not started.

BB should be initiated immediately [4,5,17] to control the signs and symptoms caused by excessive beta-adrenergic stimulation [4-6,17]. Propranolol is often the first choice [4,5] because, in addition to the BB effect [5,6], it inhibits the conversion of T4 into T3 when used in high doses [4-6,8]. In this case, bisoprolol was chosen because it is cardio-selective, making it more suitable for patients with HF. Given the cardiovascular manifestations, therapy generally also includes diuretics [10,15] (in this case, loop diuretics).

About ATDs, there are two possible strategies, with overlapping efficacy and frequency of thyrotoxicosis relapses [20,21]: titrating thionamides [20,21] (used alone with a progressive dose reduction in tandem with better control of thyroid function, documented by laboratory tests every three to four weeks) [20] and BR (use of higher doses of thionamides to completely block the thyroid axis and concomitant supplementation with levothyroxine to prevent hypothyroidism) [20,21]. This approach, used in this case, offers greater stability of thyroid function, a lower risk of iatrogenic hypothyroidism or hyperthyroidism, and reduces the need for therapeutic adjustment and frequent laboratory monitoring [20]. However, it may carry a higher risk of adverse effects, such as rash, hepatitis, or agranulocytosis [21]. In this case, no adverse effects were observed.

The recommended duration for ATDs is 12 to 18 months in GD [20,21]. With clinical improvement, it is recommended to gradually discontinue iodine solutions and corticosteroids [5,6] and adjust the doses of BB and thionamides [5,6]. The definitive therapy includes total thyroidectomy or treatment with radioactive iodine [3-6,10,15,17,20], depending on the clinical factors and/or patient preference [4,20]. In this case, radioactive iodine was selected due to the history of poor adherence to treatment and outpatient follow-up, the availability of this treatment at the hospital center, and the possibility of keeping isolation measures during the hospitalization, being for this patient a faster solution than surgery. Furthermore, some studies defend that for patients with liver disease, as in this case, radioactive iodine must be the preferred therapy, showing significant percentages of CLD remission after the treatment [12]. The cost-effectiveness and the lack of visible scars are other advantages; however, the possibility of needing lifelong TH supplementation is an important point to consider [20]. In this case, the patient reached a euthyroid state four to six months after radioactive iodine and is now on daily levothyroxine supplementation. After correcting thyroid dysfunction, a progressive resolution of the cardiovascular and hematologic alterations was observed, as well as stability of liver changes, for which the patient maintains a regular follow-up. This case illustrates a rare and advanced initial presentation of GD, involving a TS condition, in which the natural progression and systemic complications are now less frequent in developed countries due to early diagnosis and treatment. This highlights the importance of recognizing the multisystemic manifestations of TS to ensure effective therapeutic management and the prevention of complications.

## Conclusions

This article reveals how GD, which we often deal with on a daily basis and in outpatient clinics, can lead to serious manifestations, particularly when it presents itself as TS. Early recognition of symptoms and triggers is crucial to establish the correct diagnosis and to quickly start treatment and supportive measures, which have a considerable impact on the mortality rate. The main objective is to rebalance thyroid function in order to control the manifestations, which may resolve after the euthyroid state is reached, although the recovery time can be variable. The therapeutic options for the acute phase (thionamides, iodine solutions, glucocorticoids, and beta-blockers) and definitive therapies (thyroidectomy and radioactive iodine) must be chosen according to each patient's profile. This case's features, such as the long evolution of GD, the multiorganic dysfunction (cardiovascular, hepatic, and hematologic), and the poor adherence to treatment, were challenging and led to particular choices in this patient's management, such as the use of radioactive iodine during the hospital stay in order to achieve a more rapid and definitive control of GD. Regular follow-up for clinical and laboratory control and therapy adjustment was maintained after hospital discharge, with multiple complementary diagnostic tests confirming the reach of the euthyroid state and the complete resolution of cardiovascular, hepatic, and hematologic conditions within a year.

## References

[1] Taylor PN, Albrecht D, Scholz A, Gutierrez-Buey G, Lazarus JH, Dayan CM, Okosieme OE (2018). Global epidemiology of hyperthyroidism and hypothyroidism. Nat Rev Endocrinol.

[2] Wafa B, Faten H, Mouna E, Fatma M, Mohamed A (2020). Hyperthyroidism and hepatic dysfunction: report of 17 cases. JGH Open.

[3] Yorke E (2022). Hyperthyroidism and liver dysfunction: a review of a common comorbidity. Clin Med Insights Endocrinol Diabetes.

[4] De Almeida R, McCalmon S, Cabandugama PK (2022). Clinical review and update on the management of thyroid storm. Mo Med.

[5] Ross DS (2025). Thyroid storm. UpToDate.

[6] Pokhrel B, Aiman W, Bhusal K (2025). Thyroid storm. StatPearls [Internet].

[7] Barra MI, Olmos R, Barrera F, Mosso L, Domínguez JM (2020). Cholestasis secondary to hyperthyroidism in Graves' disease. Report of one case. Rev Med Chil.

[8] (2022). Japan Thyroid Association. https://www.japanthyroid.jp/en/guidelines.html.

[9] Burch HB, Wartofsky L (1993). Life-threatening thyrotoxicosis. Thyroid storm. Endocrinol Metab Clin North Am.

[10] Khan R, Sikanderkhel S, Gui J (2020). Thyroid and cardiovascular disease: a focused review on the impact of hyperthyroidism in heart failure. Cardiol Res.

[11] Ross DS, Cooper DS, Mulder JE (2025). Overview of the clinical manifestations of hyperthyroidism in adults. UpToDate.

[12] Wang R, Tan J, Zhang G, Zheng W, Li C (2017). Risk factors of hepatic dysfunction in patients with Graves' hyperthyroidism and the efficacy of 131iodine treatment. Medicine (Baltimore).

[13] Hull K, Horenstein R, Naglieri R, Munir K, Ghany M, Celi FS (2007). Two cases of thyroid storm-associated cholestatic jaundice. Endocr Pract.

[14] Lin TY, Shekar AO, Li N, Yeh MW, Saab S, Wilson M, Leung AM (2017). Incidence of abnormal liver biochemical tests in hyperthyroidism. Clin Endocrinol (Oxf).

[15] Osuna PM, Udovcic M, Sharma MD (2017). Hyperthyroidism and the heart. Methodist Debakey Cardiovasc J.

[16] Klein I, Ross DS, Mulder JE (2025). Cardiovascular effects of hyperthyroidism. UpToDate.

[17] Ross DS, Cooper DS, Mulder JE (2025). Graves' hyperthyroidism in nonpregnant adults: overview of treatment. UpToDate.

[18] Givertz MM, Borlaug BA, Dardas TF (2025). Clinical manifestations, diagnosis, and management of high-output heart failure. UpToDate.

[19] Naji P, Kumar G, Dewani S, Diedrich WA, Gupta A (2013). Graves' disease causing pancytopenia and autoimmune hemolytic anemia at different time intervals: a case report and a review of the literature. Case Rep Med.

[20] Francis N, Francis T, Lazarus JH, Okosieme OE (2020). Current controversies in the management of Graves' hyperthyroidism. Expert Rev Endocrinol Metab.

[21] Abraham P, Avenell A, Park CM, Watson WA, Bevan JS (2005). A systematic review of drug therapy for Graves' hyperthyroidism. Eur J Endocrinol.

